# Modulation of Oxygen Vacancies on Cu/ZnO by Promoter for Higher Alcohol Synthesis From Syngas

**DOI:** 10.1002/open.70233

**Published:** 2026-05-25

**Authors:** Min Tian, Enjuan Ma, Xing Tian, Wei Huang, Shirong Li

**Affiliations:** ^1^ Department of Chemistry and Chemical Engineering Taiyuan Institute of Technology Taiyuan China; ^2^ Student Affairs Department Taiyuan University Taiyuan China; ^3^ Key Laboratory of Applied Ecology of Loess Plateau College of Life Science Yan’an University Yan’an China; ^4^ State Key Laboratory of Clean and Efficient Coal Utilization Taiyuan University of Technology Taiyuan China

**Keywords:** CO hydrogenation, CuZn catalyst, higher alcohol synthesis, oxygen vacancy, promoters

## Abstract

The influence of promoters on oxygen vacancies (Ovs) within CuZn catalysts is crucial for optimizing CO hydrogenation to higher alcohols (C_2+_OH). The CuZn catalysts were successfully prepared with controlled Ovs concentrations via a fully liquid‐phase technique through the incorporation of various promoters (Cr, Al, Ga). Additional metal atoms were introduced at ZnO tetrahedral sites of Al^3+^‐ and Ga^3+^‐doped CuZn catalysts, facilitating the formation of Ovs within the lattice. Conversely, Cr formed a CuCr_2_ bimetallic phase mixture in the Cr‐doped CuZn, which thwarted the promotional effect of Cr^3+^ in the precursor and inhibited the generation of ZnO defects. Enhanced Ovs facilitated the transport of electrons from ZnO to Cu, increasing the CuZn interaction and promoting the formation of additional Cu^0^ and Zn^*δ*+^ defects. The CO dissociative intermediates (CH_x_*) were formed at Cu^0^ sites, while nondissociative intermediates (CO* and CH_*x*_O*) were formed at Cu^+^ or Ovs sites. These intermediates then participated in C—C coupling at Zn^*δ*+^ sites and subsequent hydrogenation to form C_2+_OH. The Ga‐doped CuZn catalyst showed optimal performance, yielding 17.22% CO conversion with 60.22% ethanol and 71.90% C_2+_OH proportions in the alcohol products.

## Graphical abstract

Oxygen vacancy (Ov) formation is modulated by promoter incorporation. Additional metal atoms are inserted into ZnO tetrahedral sites in CZA and CZG, thereby promoting vacancy growth. In contrast, a CuCr_2_ bimetallic phase is produced in CZC, suppressing ZnO defects. Enhanced Ovs promote ethanol formation.

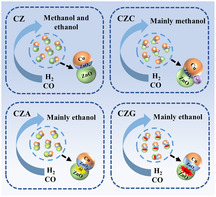

## 1 Introduction

The declining reserves of fossil fuels and clean environmental requirements have prompted the exploration of alternative energy sources [1]. Higher alcohols (C_2+_OH) have emerged as a sustainable energy alternative. They are valuable intermediates for the synthesis of various chemical products such as eco‐friendly gasoline additives and potential fuel enhancers [2]. However, existing techniques for C_2+_OH production, including petroleum‐derived alkene hydration and biological fermentation, are challenging owing to low efficiency, complicated methods for product separation, and increased raw material prices [3]. Thus, catalytic C_2+_OH synthesis from syngas‐based feedstock, including biomass, organic waste, and natural gas, is an efficient and environmentally benign approach for energy resource utilization, attracting substantial attention. While the production of C_2+_OH from syngas using CuZn catalysts is promising, owing to the economical feedstock and low energy demand, it is highly challenging because of high methanol production during the conversion [4].

A CuZnAl catalyst previously prepared via the completely liquid phase (CLP) technique exhibited an exceptional ability to produce ethanol without adding Fischer–Tropsch elements or alkali metals, thus challenging the traditional view that CuZnAl catalysts are only active for methanol synthesis [5]. However, achieving both a high ethanol ratio within total alcohols and a high overall alcohol selectivity remains challenging, as an increase in ethanol proportion typically corresponds to a decrease in total alcohol selectivity, and vice versa. Extensive studies have focused on modulating Cu, the primary active component in the catalysts, through various methods to address this challenge: introducing different additives [6], adjusting the Cu^0^/(Cu^0^+Cu^+^) ratio [7], changing the Cu particles’ size [8], and investigating the impact of AlOOH obtained via CLP on ethanol synthesis [9]. However, the impact of ZnO, an auxiliary active constituent of the catalyst, has been less explored. ZnO, a group II–VI semiconductor, often deviates from stoichiometry as a result of intrinsic defects, including vacancies in Zn and oxygen [10]. Recent studies have revealed the catalytic effect of oxygen vacancies (Ovs) on ZnO. Bai et al. [11] prepared a series of Ce‐doped activated carbon‐supported Co catalysts and explored the influence of Ovs for the direct conversion of syngas to C_2+_OH. They reported that Ovs facilitated the nondissociative adsorption and insertion of CO, involving the generation of intermediates (HCOO*, OH*, and CH_*x*_O*). Hinrichsen et al. [12] reported ZnO catalytic activity with varying Ovs; these vacancies were reported to function as active sites for hydrogenating CO to methanol. Chen et al. [13] suggested that increased oxygen defects in the CuZn catalytic system were beneficial for charge transfer and electron rearrangement, modulating the electronic characteristics at the interface of metal oxides and improving the interaction between ZnO and Cu. Ovs's catalytic activity has not yet been determined.

Numerous studies have concentrated on modulating the Ovs concentration of ZnO by incorporating various promoters. These promoters in CuZn catalytic systems exhibit two key functions: (i) enhance the catalyst's textural characteristics by adding more active sites and (ii) make sure the active phase remains evenly distributed and alters the defect structure of ZnO, which enhances the overall catalytic performance [14]. Behrens et al. [15] highlighted that modifying ZnO by partially substituting Zn^2+^ with Ga^3+^ could enhance the ZnO defect sites and interact with adjacent Cu nanoparticles, slightly changing Cu's electronic structure. This influences Cu/ZnO's active metal–support interaction, increasing catalytically active sites. The incorporation of Al to CuZn increases the number of Ovs within the ZnO lattice. These Al‐induced Ovs facilitate the dynamic movement of the ZnO_*x*_ layer under reducing conditions, covering a portion of the catalyst surface with ZnO_*x*_ and encouraging an effective interaction between ZnO and Cu. The extent of ZnO_*x*_ coverage on the Cu surface is influenced by ZnO reducibility, which can be modulated using Al‐induced Ovs, further supporting the electron‐promoting effect of Al on CuZn [16]. Similarly, the addition of Cr^3+^ to CuZn can alter the electronic structure and reducibility of ZnO, affecting Cu/ZnO interactions and catalytic activity [17]. Our primary objective was to develop promoter‐modified CuZn catalysts using the CLP method to elucidate the role of Ovs in the syngas conversion to ethanol (C_2+_OH).

Herein, a series of CuZn catalysts modified using Cr, Al, and Ga promoters was synthesized via the CLP method. These promoters successfully altered the catalysts’ as‐prepared Ovs concentration, which affected the production of alcohol. The impact of promoters on Ovs was analyzed. To further understand the chemical process involved in producing C_2+_OH from syngas, diffuse reflectance infrared Fourier transform spectroscopy (DRIFTS) in situ was utilized, offering important insights into the intermediates and active sites involved in the process.

## 2 Experimental

### 2.1 Catalyst Preparation

Cr‐, Al‐, and Ga‐doped CuZn catalysts (CZC, CZA, and CZG) were produced through the CLP procedure. Initially, citric acid (6.41 g) and the desired quantity of the promoter were dissolved in deionized water (90 mL) and stirred continuously for a duration equal to 3 h at a temperature equaling 323 K. The resulting solution was then heated for 30 min to a temperature equal to 368 K. Subsequently, a solution of Zn(NO_3_)_2_ · 6H_2_O and Cu(NO_3_)_2_ · 3H_2_O in ethylene glycol was introduced dropwise to the promoter–containing mixture with continuous agitation until a green sol developed. The obtained sol was then aged for a duration of 10 days. The aged slurry catalyst was heated for 8 h at 573 K in a nitrogen environment after being fully dispersed in liquid paraffin (300 mL).

### 2.2 Catalytic Analysis

An evaluation of the catalytic efficiency was conducted inside a 0.5 L reactor with a continuous‐flow slurry bed, where 28.92 g of catalyst was dispersed within liquid paraffin (300 mL). The catalysts were used without any pre‐reduction step. Syngas was allowed to enter the reactor at a flow rate equal to 150 mL min^−1^, maintaining a ratio of n(H_2_)/n(CO) of 2. The gas hourly space velocity was kept at 311.2 mL h^−1^· g_cat_^−1^. The reactions were performed at 4.0 MPa and 553 K with continuous stirring to ensure homogeneous mixing. The obtained products were characterized via a Haixin GC‐950 gas chromatograph. The produced gases (C_1_–C_5_ hydrocarbons, ethanol, methanol, and dimethyl ether) and H_2_, CO_2_, CH_4_, and CO were identified using a flame ionization detector (FID) and thermal conductivity detector, respectively. Liquid products obtained over a 24 h period were examined offline with an FID. The CO conversion (*X*_CO_) and product selectivity (*S* *_i_*) were estimated via Equations (1) and (2) given below

(1) $$X_{\mathrm{CO}}=\frac{F_{\text{inlet}}-F_{\text{outlet}}}{F_{\text{inlet}}}\cdot 100\%$$

(2) $$S_{i}=\frac{C_{i}\times n_{i}}{\sum C_{i}\times n_{i}}\cdot 100\%$$

where *F* represented the molar flow of CO in the gases, *C*_*i*_ indicated the molar fraction of product *i* (CH, CO_2_, ROH, and DME), and *n*_*i*_ was the carbon number of product *i*.

### 2.3 Catalyst Characterization

The crystalline phases of the catalysts were analyzed utilizing a MiniFlex II X‐ray diffractometer under continuous scanning within a 2*θ* range of 5°–85° at 8° min^−1^. The textural characteristics were examined via a Quantachrome QDS‐30 physical adsorption analyzer (USA). The Brunauer–Emmet–Teller (BET) and Barrett–Joyner–Halenda (BJH) equations were utilized to determine the pore volume (*V*_p_), specific surface area (*S*_BET_), and pore size (*D*_p_). The metal content was determined via inductively coupled plasma optical emission spectroscopy (ICP‐OES). The surface area (*S*_Cu_) and dispersion (*D*_Cu_) of Cu were measured using a chemical adsorption device based on N_2_O. The 0.1 mg catalyst was reduced for 2 h at a temperature equal to 300°C with a 10% H_2_/Ar flow rate. The determination of the hydrogen consumption was carried out while employing a temperature‐programmed reduction (TPR) analyzer (denoted as *X*). Following reduction, the catalysts were cooled down to ambient temperature after being purged with He, followed by their exposure to a 1% N_2_O/He mixture for 1 h. Subsequently, the purging was performed again to remove the remaining N_2_O. The TPR program was initiated, and the consumption of H_2_ was termed *Y*. *S*_Cu_ (m^2^·g^−1^) and *D*_Cu_(%) were obtained via Equations (3) and (4) given below

(3) $$S_{\mathrm{Cu}}=\frac{D_{m}}{\mathrm{MW}}\cdot 6.02\cdot 10^{23}\cdot \sigma_{m}\cdot 10^{-18}$$

(4) $$D_{\mathrm{Cu}}=\frac{2Y}{X}\cdot 100\%$$

where *N*_A_ denotes the Avogadro constant (6.02 × 10^23^ atom^−1^·mol^−1^), *σ*_*m*_ represents the cross‐sectional area of the Cu atoms, and MW denotes the atomic weight (g mol^−1^) of Cu. Electron paramagnetic resonance (EPR) tests were performed on a Bruker EMXplus‐6/1 spectrometer (Germany) to investigate the presence of unpaired electrons. The Raman curves were obtained via a LabRAM HR Evolution spectrometer integrated with a 325 nm laser source, a 50 × telephoto lens, and a twofold scan frequency. Using a Tianjin Xianquan TP‐5000 device, the samples’ reducibility was assessed. The Cu/ZnO interfacial areas, lattice fringes, and active site morphology were analyzed through transmission electron microscopy (TEM) and high‐resolution TEM (HR‐TEM) utilizing a Philips JEM‐2100F instrument. Surface elemental composition and electronic states were determined via Auger electron spectroscopy (AES) and X‐ray photoelectron spectroscopy (XPS) on a Thermo Scientific K‐Alpha spectrometer equipped with an Al Kα radiation source (*hν* = 1486.6 eV), operating at 12 kV with a 6 mA filament current and a 400 μm spot size. The charge correction was calibrated using the C 1*s* peak at 284.6 eV. Surface reaction intermediates were analyzed utilizing a deuterated triglycine sulfate detector fitted to a Bruker Vertex‐70 infrared spectrometer. (It is important to note that while this work uses the same synthesized materials as our previous study on Ga content [https://doi.org/10.1021/acssuschemeng.3c03091], here we focus on an entirely different aspect: the role of structural promoters. This independent variable and its underlying mechanism have not been previously discussed.)

## 3 Results and Discussion

### 3.1 XRD Analysis

**FIGURE 1:**
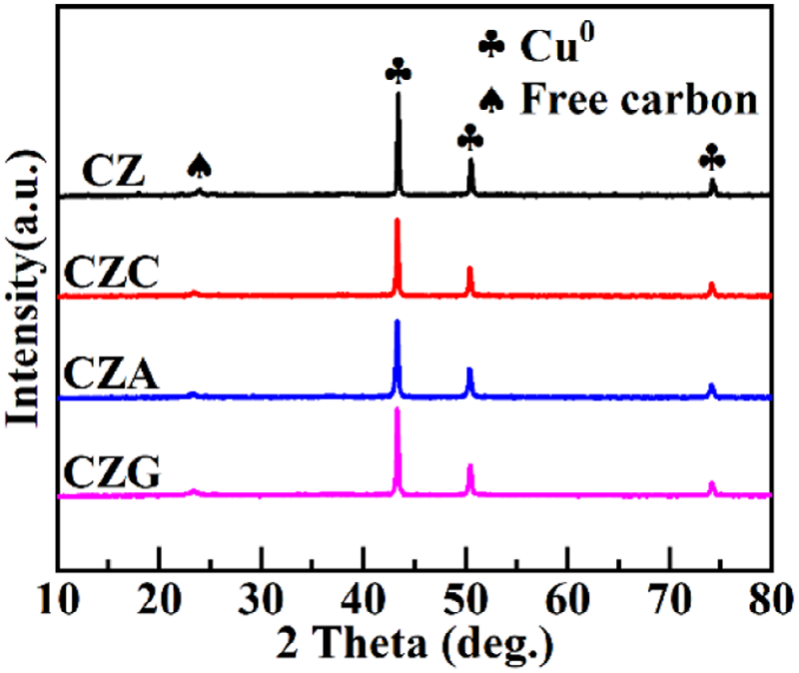
The XRD profiles obtained for the catalysts.

CZC, CZA, and CZG were analyzed via XRD to study their crystal structures. Figure 1 shows that the as‐prepared catalysts displayed structures with similar characteristics, suggesting that the incorporated promoters have a minimal impact on the catalyst crystal structures. Distinct diffraction signals at 43.4°, 50.5°, and 74.1° were ascribed to the planar directions (111), (200), and (220) of metallic Cu^0^, respectively, and are characteristic of the CLP synthesis process [18]. The disappearance of ZnO diffraction peaks suggests that ZnO is present in an amorphous or highly dispersed form. The weak diffraction signal at 23.6° corresponds to carbon, arising from the decomposition of liquid paraffin [19]. Furthermore, the lack of the Cr, Al, and Ga diffraction peaks suggested that these promoters are either incorporated or highly dispersed within the ZnO matrix. The mean particle size of Cu before the reaction (*d*_Cu_) was determined via Scherrer's equation, and the findings are summarized in Table 1.

**TABLE 1:** Physicochemical characteristics of CZ, CZC, CZA, and CZG.

Catalyst	aCu, wt.%	aZn, wt.%	aM, wt.%	M/(M + Cu + Zn), mol mol^−1^	b *S*_BET_, m^2^·g^−1^	b *V*_P_, cm^3^·g^−1^	b *D*_P_, nm	c *d*_Cu_, nm
CZ	81.38	18.62	—	—	45.74	0.18	11.32	30.5
CZC	74.51	19.98	5.51	0.055	62.28	0.22	14.00	29.8
CZA	73.05	21.19	5.77	0.058	64.21	0.21	14.53	32.6
CZG	74.88	19.47	5.65	0.057	64.35	0.24	15.16	26.5

a Metal loading amounts measured via ICP.

b *S*_BET_, *V*_p_, and *D*_p_ determined via N_2_ physical adsorption.

c The Scherrer equation was utilized to get the average Cu particle size (nm).

### 3.2 Analysis of N_2_ Desorption/Adsorption Isotherms

**FIGURE 2:**
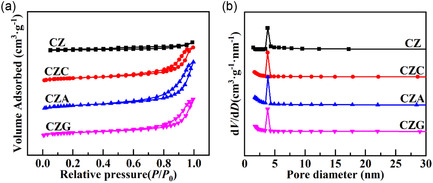
(a) The N_2_ desorption/adsorption isotherms and (b) pore size distributions obtained for CZ, CZC, CZA, and CZG.

The pore size distributions and the N_2_ desorption/adsorption isotherms were used to examine the textural properties of CZM (Figure 2). With H3 hysteresis loops and a Type IV classification, the isotherms showed a mesoporous structure with slit‐shaped pores generated by lamellar particle stacking [20]. Notably, each catalyst displays a uniform pore configuration with an estimated 3.8 nm pore size. The textural characteristics of the catalysts, including *S*_BET_, *V*_p_, and *D*_p_, are compiled in Table 1. According to these results, the textural characteristics of catalysts with excess mesopores can be improved by adding a sufficient amount of promoters, which will facilitate contact between reactant molecules and the surface‐active species. Besides, Table 1 presents the actual composition of the catalyst's active components. The measured ratios closely match the designed feed ratios, confirming that the preparation process is efficient and involves minimal loss of active materials.

### 3.3 N_2_O Chemisorption Analysis

The *S*_Cu_ and *D*_Cu_ of N_2_O chemisorption are presented in Table 2. The distinct structures of the promoters effectively modulate the *S*_Cu_ of the CuZn catalyst, and the order of modulation of the catalysts is CZA < CZ < CZC < CZG. However, the order varies based on the variation of the Cu particle size. A large *S*_Cu_ is exposed by small Cu particles, improving catalytic efficiency and showing a linear relationship with catalytic activity. High *S*_Cu_ causes extra H atoms to dissociate from the H_2_ adsorbed by the Cu sites, accelerating the adsorbed carbon‐based species’ hydrogenation [21]. A similar variation exists between *D*_Cu_ and CO conversion, indicating that finely dispersed Cu species are essential for increasing Cu‐based catalysts’ catalytic activity [22].

**TABLE 2:** N_2_O chemisorption analysis.

Catalyst	a *S*_Cu_, m^2^·g^−1^	a *D*_Cu_, %
CZ	36.10	5.6
CZC	46.42	7.2
CZA	32.88	5.1
CZG	70.27	10.9

a *S*_Cu_ and *D*_Cu_ obtained from N_2_O chemisorption.

### 3.4 EPR Analysis

**FIGURE 3:**
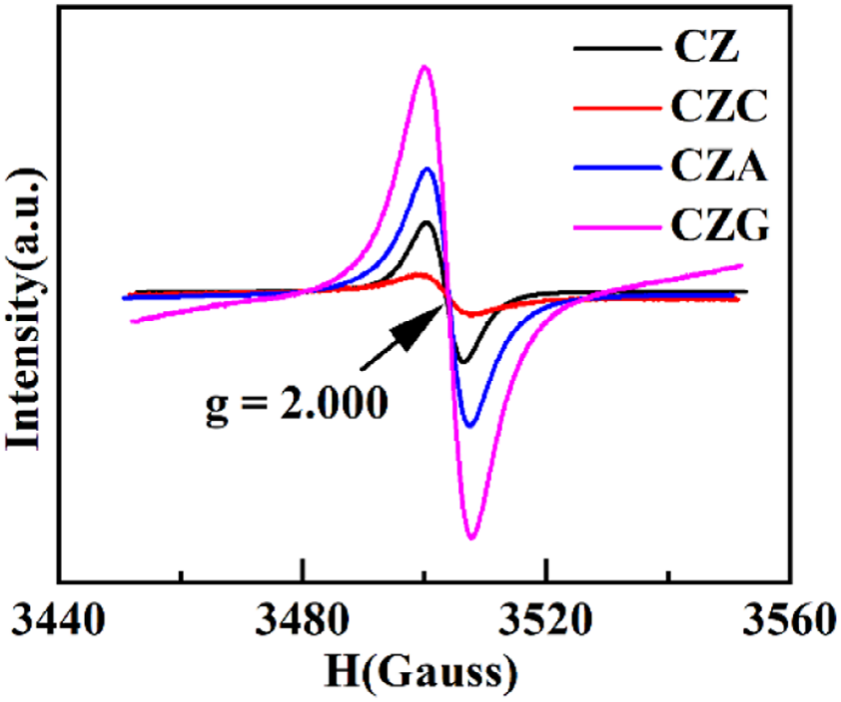
EPR profiles of CZ, CZC, CZA, and CZG.

The effect of Ovs sites on C_2+_OH synthesis from syngas was examined via EPR. It can be seen in Figure 3 that CZC, CZA, and CZG exhibited EPR signals at the g value equal to 2.000, signifying the presence of unpaired electrons and confirming the existence of Ovs within the catalysts [23]. The Ovs sites on the ZnO surface act as electron traps, facilitating the adsorption of reactive species and regulating charge transfer between the adsorbates and the catalyst surface. These processes influence the decomposition and formation of reaction intermediates, therefore influencing the product's selectivity as well as the rate of reaction [24]. Furthermore, the intensity of the EPR signal correlates with the concentration of Ovs; a stronger signal reflects a higher Ovs content. The relative Ovs concentration of the catalysts followed the order CZG > CZA > CZ > CZC. Quantitative data presented in Table 3 corroborate the above analysis.

**TABLE 3:** Ovs content of CZ, CZC, CZA, and CZG.

Catalyst	Ovs (10^16^ spins·g^−1^)
CZ	3.6
CZC	3.1
CZA	4.6
CZG	5.5

### 3.5 Raman Analysis

**FIGURE 4:**
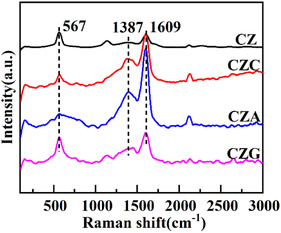
Raman profiles of CZ, CZC, CZA, and CZG.

The molecular structure of CZM was examined via Raman spectroscopy. As shown in Figure 4, no characteristic vibrational modes of the Cu—O—Cu or Cu—O bonds were observed at 295, 342, or 627 cm^−1^, confirming that the catalyst lacks a CuO phase. The signal at 567 cm^−1^ represents the Zn‐O longitudinal optical mode, specifically the *A*_1_(LO) band, representing internal defects such as Ovs. Moreover, it is directly proportional to the Ovs concentration [25]. Hence, an increase in Ovs proportionally increases the *A*_1_(LO) band intensity. According to the EPR analysis, the Ovs content follows the order CZG > CZA > CZ > CZC. Further, two prominent signals at 1387 and 1609 cm^−1^ correspond to the D band, arising from defects, and the G band, linked to the ordered graphite arrangement. The D band is linked to disordered regions in the graphite layer and defects resulting from lattice mismatch. The G peak represents an *E*_2g_ mode characterized by the stretching vibration of *sp* ^2^ C—C bonds [8].

### 3.6 H_2_‐TPR Analysis

**FIGURE 5:**
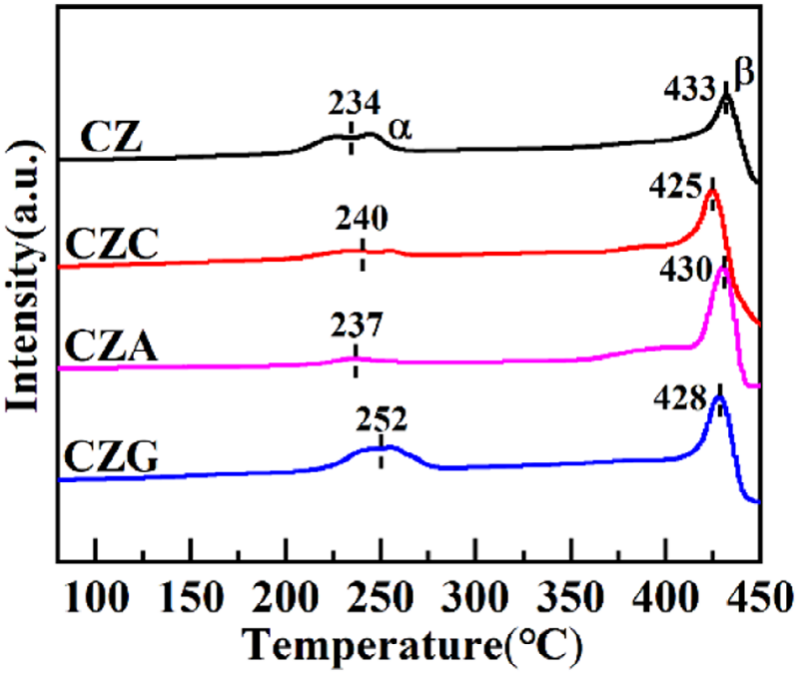
H_2_‐TPR profiles of CZ, CZC, CZA, and CZG.

H_2_‐TPR was employed to investigate the redox behavior of the metal oxides. As illustrated in Figure 5, the reduction profiles of CZ, CZC, CZA, and CZG display peaks between 230°C and 450°C. These peaks can be categorized based on their temperature positions into a low‐temperature *α* signal (230°C–255°C) and a high‐temperature *β* signal (425°C–435°C). The *α* peak implied the reduction of highly dispersed Cu^+^, while the *β* peak indicated the Cu^+^ ions that interacted with ZnO [26]. Table 4 presents the amount of H_2_ consumed corresponding to the *β* peak during reduction. The catalysts’ H_2_ consumption order is CZG > CZA > CZ > CZC, indicating that the Ga‐doped CuZn catalyst effectively improves the reducibility of Cu^+^ interacting with ZnO and enhances the Cu/ZnO interaction [27].

**TABLE 4:** H_2_ consumption by CZ, CZC, CZA, and CZG.

	H_2_ consumption, mmol g^−1^	
Catalyst	*α*	*β*
CZ	0.070	0.121
CZC	0.017	0.118
CZA	0.085	0.154
CZG	0.031	0.164

### 3.7 TEM Analysis

**FIGURE 6:**
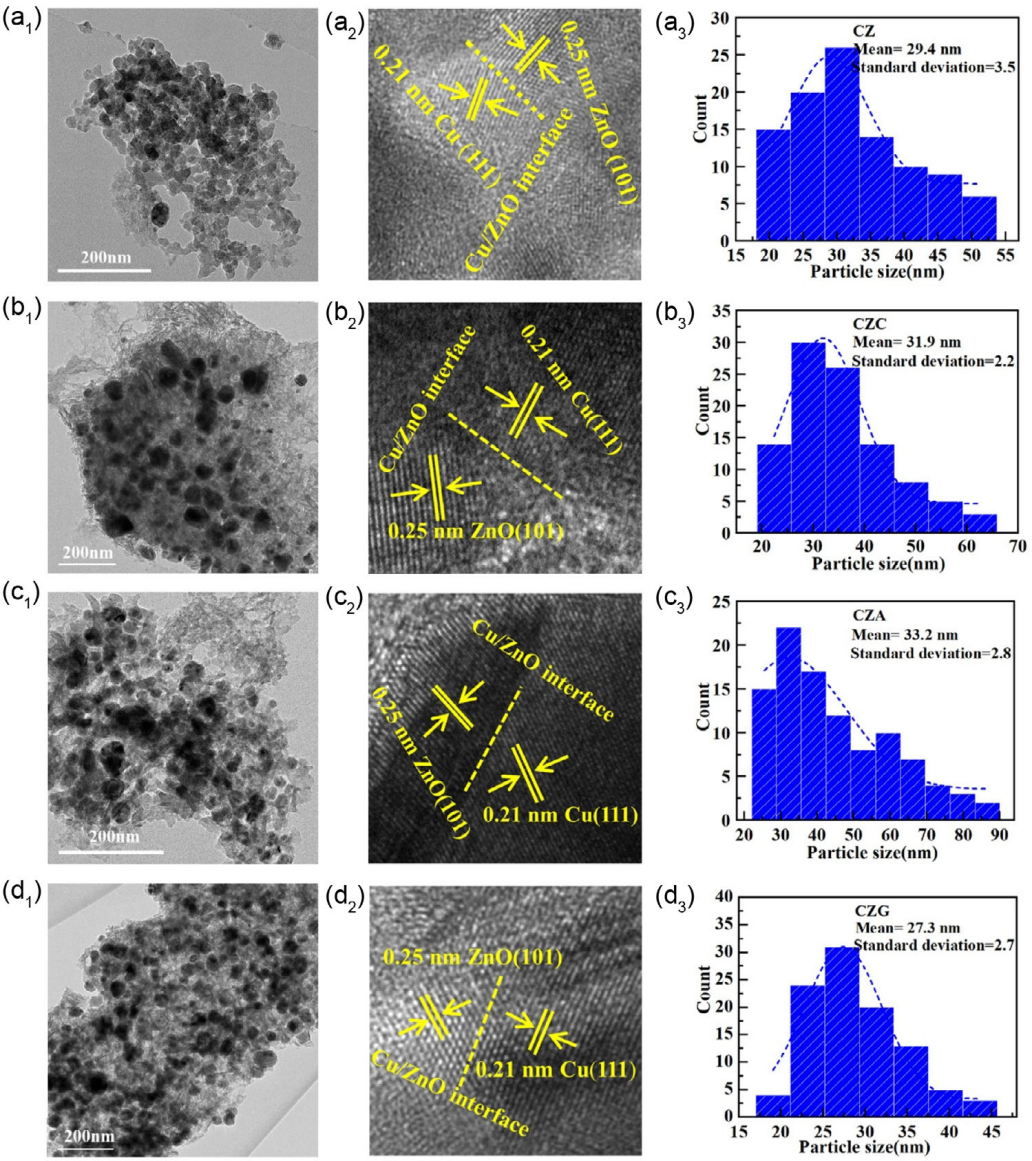
The (a_1_–d_1_) The TEM and (a_2_–d_2_) HRTEM images, along with (a_3_–d_3_) size distributions of the Cu particles in (a_1_,a_2_,a_3_) CZ, (b_1_,b_2_,b_3_) CZC, (c_1_,c_2_,c_3_) CZA, and (d_1_,d_2_,d_3_) CZG.

The Cu particle size in the catalysts was further evaluated using TEM. Figure 6a_3_–d_3_ shows that the average Cu particle sizes for CZ, CZC, CZA, and CZG are 29.4, 31.9, 33.2, and 27.3 nm, respectively. These findings suggested that the incorporation of various promoters significantly affected the particle size of Cu, consistent with the variations observed in the XRD analysis. Furthermore, the size of Cu particles determined via TEM is larger than that determined via XRD. The exposed surface of Cu particles detected via TEM comprises the Cu (111) surface. The Cu grain size data provide a comprehensive average of all crystallographic planes: (111), (200), and (220) [8]. Figure 6a_2_–d_2_ presents the high‐resolution HRTEM image of CZ, CZC, CZA, and CZG. Amplification of the dark area differentiated the crystal planes ZnO (101) and Cu (111) with interplanar separations of 0.25 and 0.21 nm, respectively [28]. The interface between ZnO and Cu displayed a strong interaction between the two components, promoting the active catalytic sites’ formation and enhancing CO hydrogenation to C_2+_OH [29].

### 3.8 XPS Analysis

**FIGURE 7:**
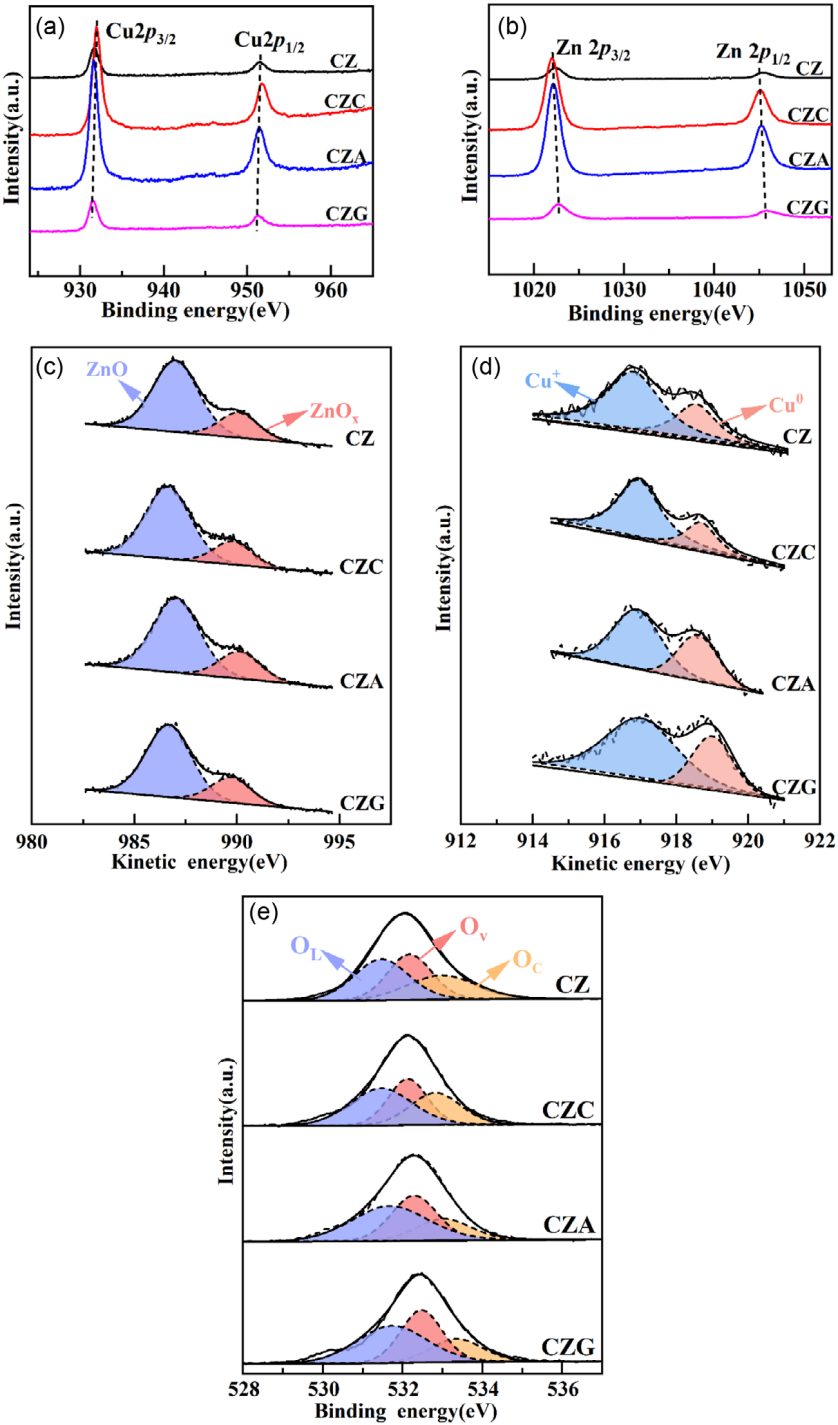
(a) The Cu 2*p*, (b) Zn 2*p*, (c) Zn LMM, (d) Cu LMM, and (e) O 1*s* XPS profiles obtained for CZ, CZC, CZA, and CZG.

Important factors throughout the reaction process include each element's electronic state, valence state distribution, and current form on the catalyst surface. Thus, the prepared catalysts were characterized using XPS and XPS‐AES. The Cu 2*p* XPS spectrum in Figure 7a shows two distinct signals between 932 and 960 eV, representing the Cu 2*p*_1/2_ and Cu 2*p*_3/2_ states at 952.6 and 932.6 eV, respectively. The absence of satellite peaks in the 940–945 eV region confirms that Cu exists predominantly as Cu^+^ and Cu^0^ [30]. The Zn 2*p* XPS spectrum is presented in Figure 7b, which shows signals at 1044.8 and 1021.7 eV associated with the Zn 2*p*_1/2_ and Zn 2*p*_3/2_ levels in ZnO, respectively [31]. The Cu 2*p* peak in CZC shifts to higher energy, whereas in CZA and CZG it shifts to lower energy relative to CZ. However, the Zn 2*p* peak shows an opposite trend. These shifts in the Cu 2*p* and Zn 2*p* provide further evidence that the concentration of Ovs in the catalysts follows the order CZG > CZA > CZ > CZC [32].

Figure 7c shows the Zn LMM AES spectrum. The signals at 990.2 and 987.0 eV were ascribed to the Zn^*δ*+^ (0 < δ < 2) and Zn^2+^ ions, respectively. The presence of the Zn^*δ*+^ ions can be linked to the partially reduced Zn^2+^ ions, which suggests that Ovs were produced during the reduction procedure [33]. Thus, the Ovs content was quantified using the Zn^*δ*+^/(Zn^*δ*+^+Zn^2+^) ratio. The catalysts’ Ovs content is arranged as follows: CZG > CZA > CZ > CZC (Table 5).

**TABLE 5:** XPS characteristics of surface elemental composition, Cu LMM, Zn LMM, and O 1*s*.

Catalyst	Zn^*δ*+^/(Zn^*δ*+^+ Zn^2+^)	Cu^0^/(Cu^0^+ Cu^+^)	*A*_OV_/(*A*_OL_ + *A*_OV_ + *A*_OC_)	Surface Composition, At.%			Molar Ratio
				Cu	Zn	M	Cu/(Zn + Cu)
CZ	0.231	0.32	34.25	34.59	65.41	—	34.59
CZC	0.213	0.26	31.73	14.39	50.56	35.05	22.15
CZA	0.242	0.37	35.29	13.53	47.67	38.80	22.10
CZG	0.245	0.39	36.52	23.59	55.98	20.43	29.65

The distribution of Cu species is pivotal for the selectivity of the products in catalytic reactions. The Cu LMM spectrum was used for peak fitting to differentiate between Cu^+^ and Cu^0^. Figure 7d shows two peaks at 916.8 and 918.6 eV, indicating Cu^+^ and Cu^0^, respectively, in the Cu LMM spectrum. The ratio of Cu^0^ to Cu^+^ was determined via peak area analysis of the spectrum. Table 5 shows that the variation in Cu^0^/(Cu^0^+Cu^+^) is similar to that in Zn^*δ*+^/(Zn^*δ*+^+Zn^2+^) and follows a similar pattern to the changes in the Ovs content. This correlation suggests that the formation of Ovs during reduction facilitates electron transfer. The Ovs facilitated the migration of electrons from ZnO to Cu, increasing Cu reducibility while generating an excess of catalytically active sites [34].

The catalysts’ O 1*s* XPS profiles display broad, asymmetric peaks that can be deconvoluted into three distinct components, reflecting different oxygen species in varying chemical environments (Figure 7e). The primary peak (531.1 eV) indicates the lattice oxygen (*O*_L_) associated with the lattice metal. The peak at 532.0 eV is ascribed to oxygen atoms adjacent to surface Ovs in the catalysts, while the peak at 532.9 eV is ascribed to surface chemisorbed oxygen (Oc), which indicates the oxygen bonded to the catalyst surface but not to the lattice structure [35]. The O 1*s* spectra were analyzed by deconvoluting the peaks corresponding to *O*_L_, Ov, and *O*_C_, and the results are presented in Table 5. Ov% was determined employing the equation Ov (%) = AOv /(AOv + AOc + AO_L_). The Ov content increases in the order CZG > CZA > CZ > CZC, in agreement with the previously discussed findings.

**FIGURE 8:**
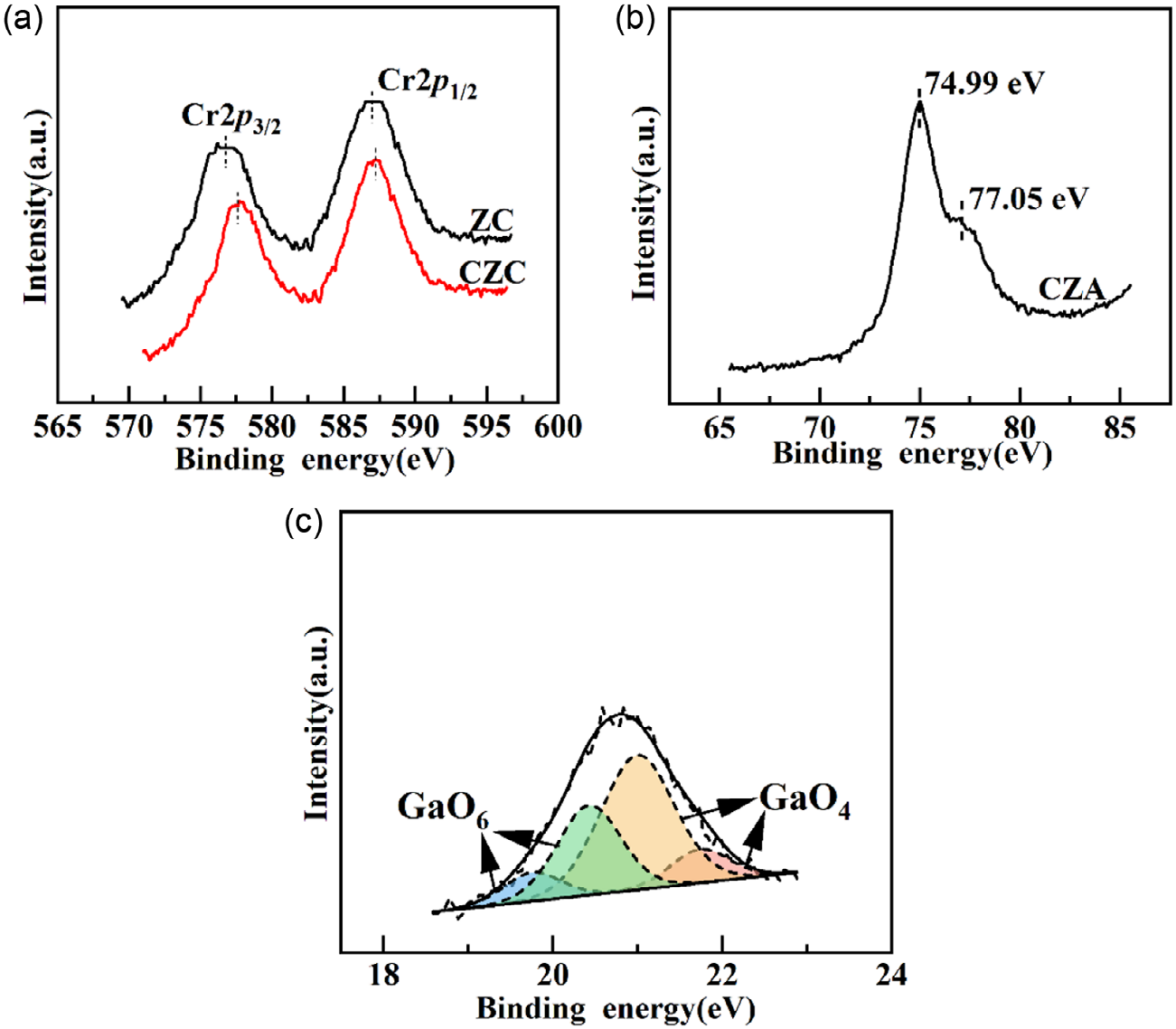
(a) The Cr 3*d*, (b) Al 3*d*, (c) Ga 3*d* XPS profiles obtained for ZC, CZC, CZA, CZG.

**FIGURE 9:**
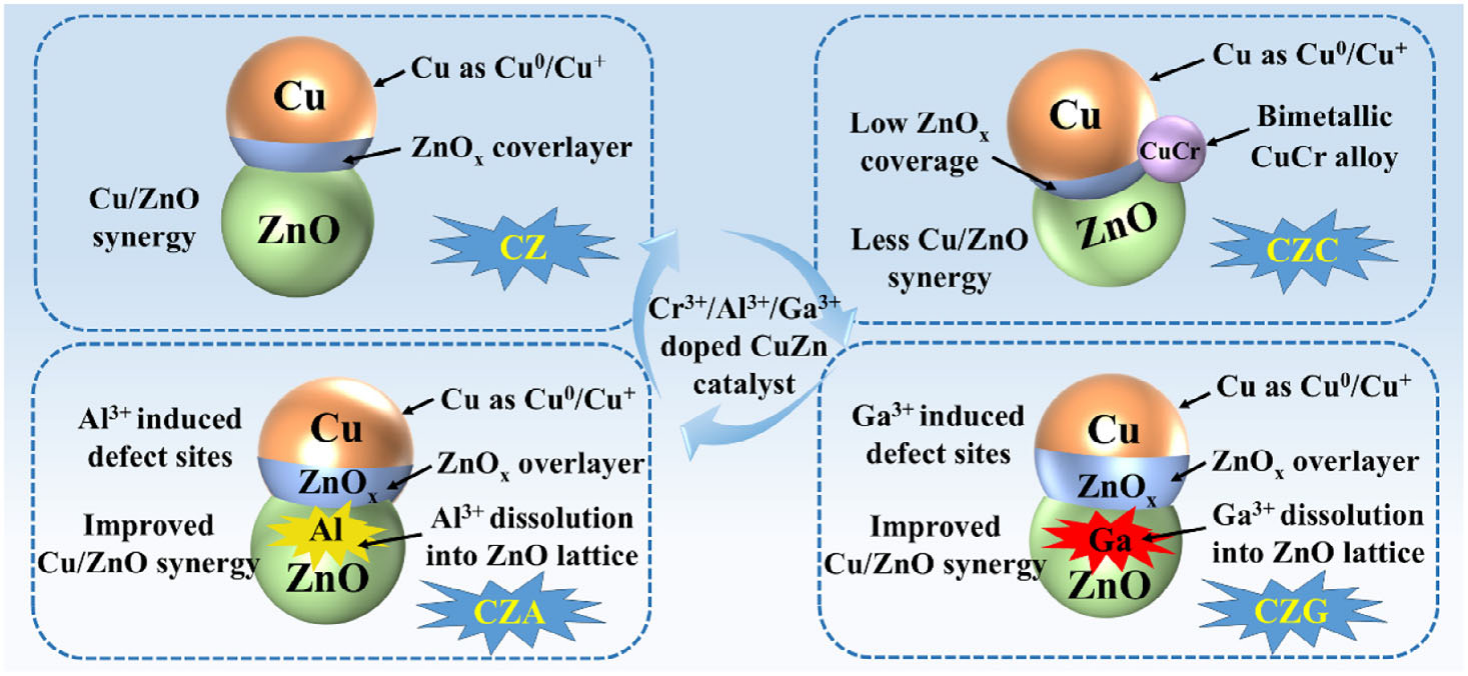
Effect of Cr, Al, and Ga doping on the structure of the CuZn catalysts.

The impact of the promoters on the CuZn catalyst structure was studied by examining their chemical states (see Figure 8a–c). The Cr 2*p* XPS spectra of ZC exhibited two distinct signals at 586.7 and 587.0 eV, corresponding to Cr 2*p*_3/2_ and Cr 2*p*_1/2_, respectively, indicating the presence of Cr^3+^ [36]. The introduction of Cu raises the binding energy of Cr 2*p* in CZC, indicating electronic interaction between Cr and Cu species and the generation of a CuCr_2_ bimetallic phase [37]. The Al 2*p* spectrum of CZA displayed signals at 77.05 and 74.99 eV, assigned to the octahedral and tetrahedral coordinations, respectively. This suggested that Al atoms were incorporated into both sites of the ZnO lattice [5]. The Ga 3*d* spectrum of CZG reveals that Ga exists in both tetrahedral ([GaO_4_]) and octahedral ([GaO_6_]) coordination, suggesting that some Ga atoms substitute Zn^2+^ to form tetrahedral sites, while others occupy octahedral interstitial positions [38]. Thus, promoters modulate the Ovs concentration in the catalysts. The introduction of Cr forms a CuCr_2_ bimetallic phase through the interaction between Cu and Cr_2_O_3_. This formation offsets the promotional effect of Cr^3+^ in the precursor and suppresses the generation of Ovs in ZnO. Alternatively, Ga^3+^ and Al^3+^ promoters integrated into the ZnO lattice's tetrahedral sites promoted the development of Ovs (Figure 9).

Table 5 presents an analysis of the elemental composition on the surface of CZ, CZC, CZA, and CZG. Comparison with the ICP analysis data reveals a decrease in surface Cu concentration accompanied by an increase in Zn content, indicating Zn enrichment at the catalyst surface. Furthermore, the promoters also accumulate on the surface due to their interactions with the catalyst components, influencing the overall catalytic performance.

### 3.9 Activity Evaluation Results

**FIGURE 10:**
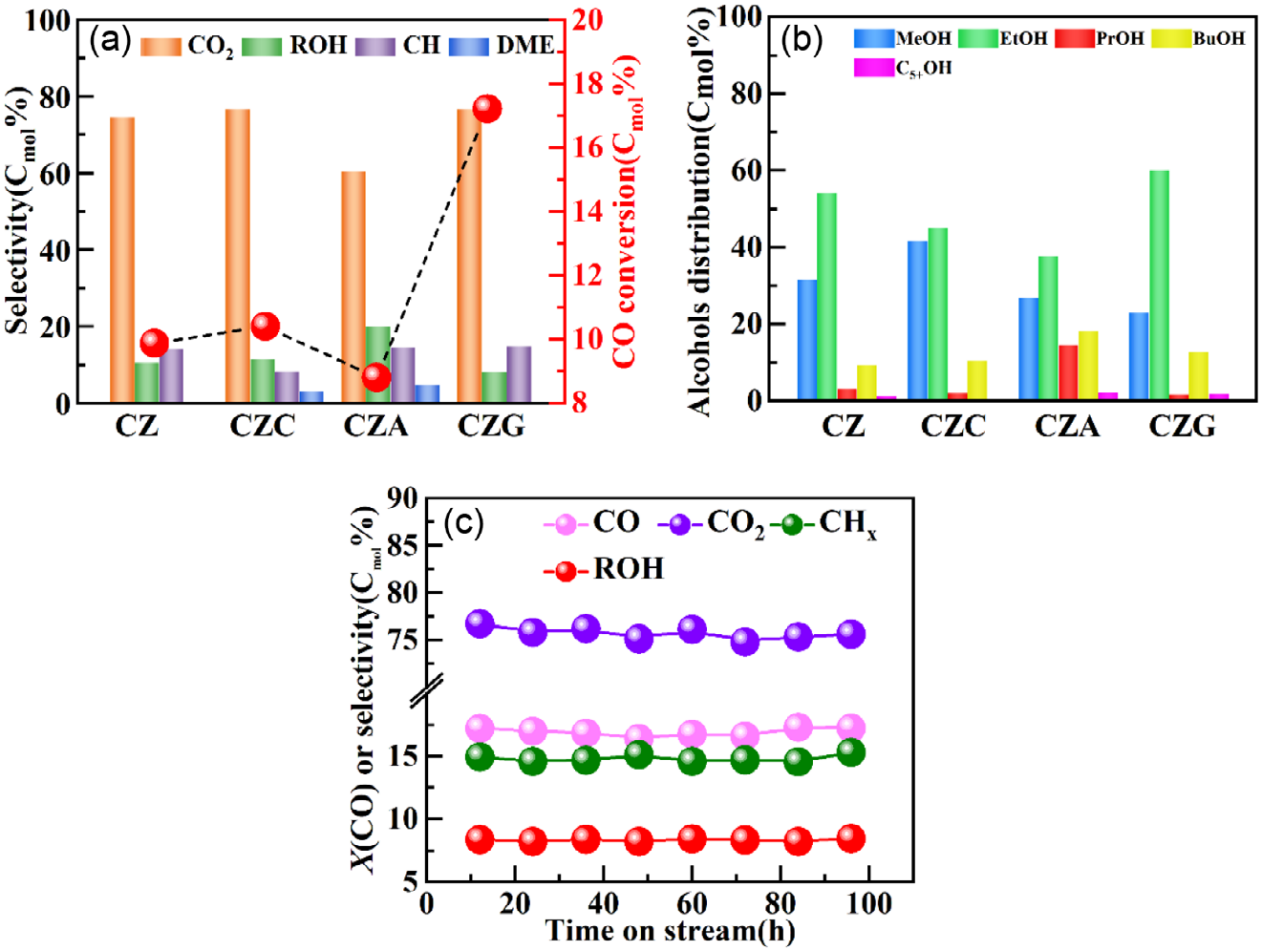
(a) CO conversion and product distribution, (b) alcohol distribution catalyzed by CZ, CZC, CZA, and CZG, and (c) CZG stability.

The activity of the as‐prepared catalysts for CO hydrogenation was studied at 4.0 MPa and 280°C, with an H_2_/CO ratio equal to 2:1. The results, as depicted in Figure 10a, show distinct differences in product distribution and catalytic activity based on the catalyst composition. The following product selectivity is catalyzed by CZ: ROH (10.66%), CO_2_ (74.93%), and CH_*x*_ (14.14%). The incorporation of Cr in CZC diminishes the catalytic activity, indicated by a reduced CH_*x*_ (8.45%), slightly elevated CO_2_ (76.75%), and heightened ROH (11.6%) selectivity. Conversely, the introduction of Al and Ga in CZA and CZG, respectively, significantly improves the catalytic performance. The Al incorporation in CZA increases the CH_*x*_ (14.59%) and ROH (20.06%) selectivity, while CO_2_ selectivity (60.45%) is decreased. The addition of Ga in CZG exhibits a highly pronounced effect, yielding the highest CO conversion of 17.22%. This enhancement is correlated with the increased surface area exposed by Cu^0^. CZG exhibits a CH_*x*_, ROH, and CO_2_ selectivity of 14.92%, 8.37%, and 76.7%, respectively. Overall, the CO_2_ selectivity is high. This is due to the active water‐gas shift reaction (WGSR) within the reaction system. During CO hydrogenation to C_2+_OH, H_2_O is inevitably generated as a byproduct. The employed Cu/ZnO‐based catalyst possesses a strongly hydrophilic surface, which readily adsorbs and activates the produced H_2_O, thereby significantly accelerating the WGSR (CO + H_2_O → CO_2_ + H_2_) and converting a substantial portion of CO to CO_2_ [24].

Figure 10b illustrates the detailed alcohol distribution. Compared with CZ, the CZC catalyst shows a significant change in product composition: the methanol fraction increases to 31.75%, while the C_2+_OH fraction decreases to 49.55%. This indicates that the presence of Cr alters the alcohol distribution by forming a CuCr_2_ bimetallic phase, which counteracts the promotional effect of Cr^3+^ in the precursor and suppresses the generation of Ovs in ZnO, negatively impacting the C_2+_OH proportion. CZA and CZG remarkably enhance C_2+_OH proportions. CZG exhibits exceptional catalytic performance, achieving high proportions of ethanol (60.22%) and C_2+_OH (71.90%). This superior performance was owing to the incorporation of Ga^3+^ into ZnO tetrahedral sites, promoting the formation of more oxygen defects. Thus, the catalyst synthesized C_2+_OH in high yield. The evaluation of the stability of CZG, as depicted in Figure 10c, demonstrates its excellent stability for 96 h. The enhanced stability and improved catalytic activity of CZG for the preparation of C_2+_OH fractions make it a promising contender for the formation of C_2+_OH from syngas.

### 3.10 Role of Ovs

**FIGURE 11:**
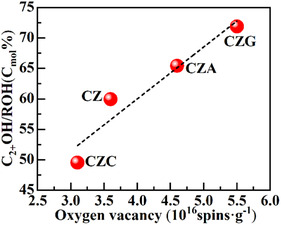
Dependency of the C_2+_OH/ROH ratio on Ovs.

Through an analysis of the correlation between the Ovs content and the C_2+_OH fraction, the impact of Ovs on C_2+_OH production was investigated (Figure 11). The findings revealed a strong linear positive correlation between the Ovs content and C_2+_OH production.

### 3.11 Potential Reaction Mechanism for Alcohol Synthesis

The CO hydrogenation mechanism to C_2+_OH over the CZM catalyst was investigated using in situ DRIFTS under a gas mixture of CO, H_2_, and Ar at 5, 10, and 15 mL min^−1^, respectively, at 553 K and 0.1 MPa (Figure 12). The band found at 1622 cm^−1^ indicated OH* species [39], while those observed at 1557 and 1390 cm^−1^ indicate HCOO* species, derived from antisymmetric *ν*_as_(OCO) and symmetric *ν*_s_(OCO) vibrations, respectively [40]. CH_*x*_O* (1683 and 1613 cm^−1^) are formed from CO and H_2_ adsorbed on Cu^0^ sites, which dissociate into O*, C*, and H* and subsequently undergo hydrogenation to generate OH*. The produced OH* interacts with the activated CO* of Ovs and forms HCOO*, which is finally hydrogenated to form CH_*x*_O* [41]. Notably, the signal intensity of CH_*x*_O* and the Ovs concentration of CZG are higher than those of others, illustrating that the high Ovs facilitates the CH_*x*_O* formation in the synthesis of alcohol.

FIGURE 12In situ H_2_ + CO DRIFT spectra across (a,b) CZ, (c,d) CZC, (e,f) CZA, and (g,h) CZG.

**Figure:**
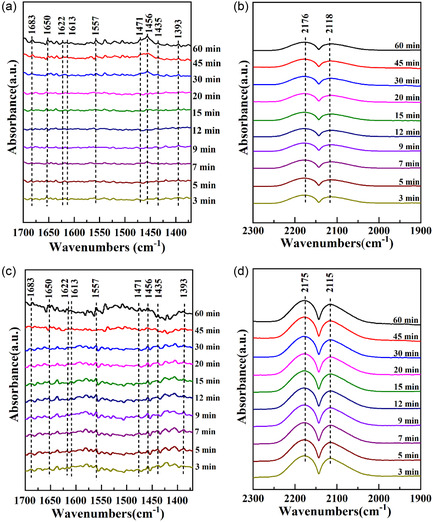

**Figure:**
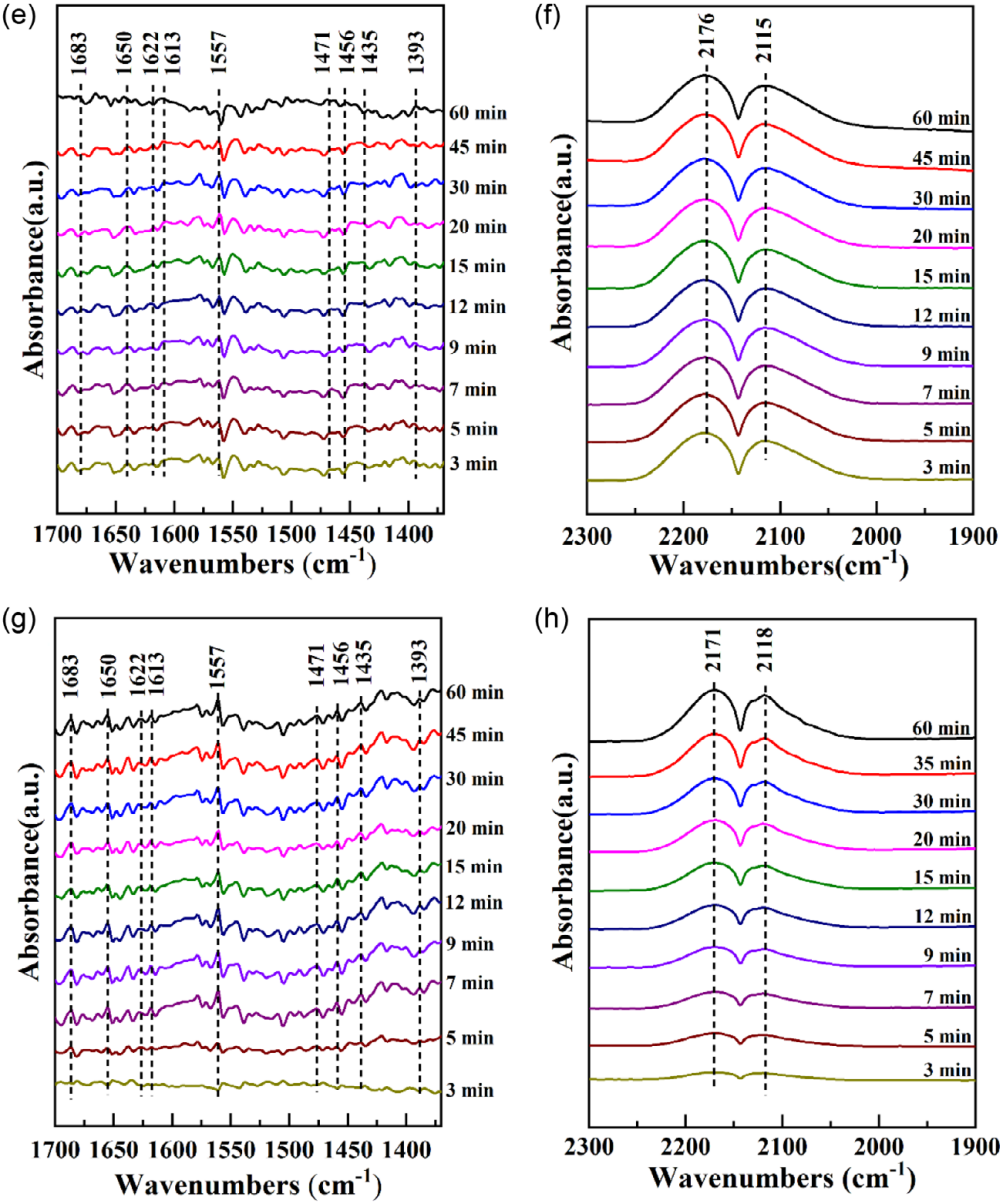

The bands appearing at 2177 and 2115 cm^−1^ corresponded to CO gas, and CO adsorbed linearly on Cu sites, respectively. CO binds more stably in a linear configuration to Cu^+^ sites compared with Cu^2+^ and Cu^0^ sites, with the signal at 2117 cm^−1^ specifically assigned to linear Cu^+^‐CO [42]. The signals at 1456 and 1471 cm^−1^ were ascribed to the stretching *ν*_s_(C—H) and C—H bending *δ*(C—H) vibrations of the CH_2_* species, respectively [41]. The CH_x_* species, which act as key intermediates in C_2+_OH formation, were generated on the Cu^0^ active sites through CO hydrogenation and dissociation or C—O bond cleavage in CH_*x*_O species. The adsorption intensity of CH_x_* on CZG was higher than that of others, indicating that CZG contained a high concentration of CH_x_*. The Cu^0^ sites serve as the active centers for CO/CH_*x*_O* species’ dissociation and hydrogenation, which aligns with the H_2_‐TPR and XPS findings showing a higher Cu^0^/(Cu^0^+Cu^+^) ratio in CZG than that of others [43].

The emergence of CH_*x*_CHO* (1434 cm^−1^) and CH_*x*_CO* (1437 cm^−1^) signals suggests that the C‐chain grows owing to the insertion of CO/CH_*x*_O* into CH_x_* species [44]. In the production of C_2+_OH, this is regarded as the rate‐determining step. Studies report a correlation between the oxygen‐deficiency species of Zn^*δ*+^ and the C‐chain growth ability. The proportion of Zn^*δ*+^ species in CZG was higher than that of others, suggesting that it possessed an enhanced capacity for C‐chain growth at Zn^*δ*+^ sites. The high concentration of Zn^*δ*+^ can effectively catalyze the insertion reactions and subsequent hydrogenation steps necessary for ethanol (C_2+_OH) formation.

**SCHEME 1:**
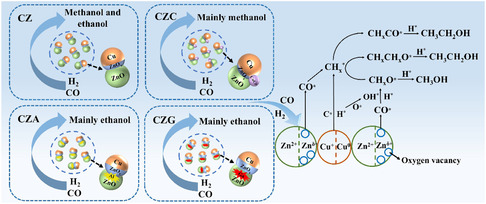
CO hydrogenation mechanism over catalysts to C_2__+_OH.

According to characterizations of different metal (M^3+^)‐doped catalysts, we propose the CO hydrogenation mechanism to C_2+_OH catalyzed by CZM (Scheme 1). The XPS results revealed that Ga^3+^ and Al^3+^ ions were included in the tetrahedral sites of the ZnO lattice during the reaction, facilitating the generation of Ovs in ZnO. However, Cr^3+^ interacts with Cu to generate a CuCr_2_ bimetallic phase, which counteracts the beneficial effect of Cr^3+^ in the precursor and suppresses defect formation in the ZnO structure. The increased Ovs facilitates the release of excess electrons that can reach the Cu surface through the Schottky barrier, strengthening the interaction between ZnO and Cu, leading to a higher Cu^0^/(Cu^0^ + Cu^+^) ratio and a Zn^*δ*+^ defect structure. Ovs activate CO to form HCOO* and CH_*x*_O* intermediates. CH_x_*, arising from partial dissociation of CH_*x*_O*, following hydrogenation, or dissociation of CO on Cu^0^ sites, engages in C—C coupling with CH_*x*_O*/CO* on Zn^*δ*+^ sites to produce ethanol (C_2+_OH).

### 3.12 Catalytic Performance Comparison

Table 6 compares the CZG catalyst developed in this work with representative catalysts for ethanol synthesis reported in the literature. Although the CZG catalyst exhibits a relatively lower ROH selectivity, it achieves an exceptionally high ethanol distribution of 60.22% among the produced alcohols, along with a CO conversion of 17.22%, highlighting its promising potential for ethanol synthesis. This feature is particularly significant when contrasted with catalysts that show high ROH selectivity but predominantly produce methanol, a product of substantially lower economic value.

**TABLE 6:** Comparison of catalytic performance between the CZG catalyst and reported catalysts.

Cat	CO Con, %	ROH Selectivity, %	ROH distribution, %					Ref.
			*C*_1_	*C*_2_	*C*_3_	*C*_4_	*C*_5_	
Cu_3.5_Zn_0.5_Al_0.4_	7.90	36.80	31.40	38.80	6.30	11.90	11.60	[8]
Co‐7.5Ce/AC	16.00	23.50	27.60	29.50	12.10	9.80	11.30	[11]
Cu_1_Al_1_	6.57	9.47	75.14	24.86	—	—	—	[45]
Cu_1_Zn_1_	3.36	91.73	97.45	1.92	—	—	—	[46]
CZG	17.22	8.37	22.99	60.22	1.79	13.00	2.00	This work

The scientific advancements of our approach are twofold. First, the high ethanol proportion suggests effective promotion of C—C coupling while suppressing methanol formation, reflecting precise control over the reaction pathway. Second, the combination of competitive CO conversion and high ethanol distribution leads to a favorable ethanol space‐time yield, demonstrating efficient synergy between the active sites responsible for CO activation and carbon chain growth. Consequently, this work achieves, for the first time, synergistic catalysis of directional C—O bond cleavage and C—C bond coupling for ethanol production from syngas over an oxygen‐vacancy‐regulated CZG catalyst.

Furthermore, our product‐oriented design, which prioritizes the distribution of the target product (ethanol) while maintaining high CO conversion, represents a more rational strategy for the synthesis of value‐added alcohols than simply pursuing ROH selectivity.

## 4 Conclusions

Three Cr‐, Al‐, and Ga‐modified catalysts, CZC, CZA, and CZG, were prepared via the CLP technique and investigated for the generation of C_2+_OH from syngas. The introduction of appropriate promoters (Cr, Al, and Ga) influenced the physical properties of the catalysts, including *S*_BET_, *V*_p_, *D*_p_, Cu^0^ size, *S*_Cu_, and *D*_Cu_. The promoters could modulate the number of Ovs. Additional metal atoms were incorporated into ZnO's tetrahedral sites in CZA and CZG, promoting the growth of Ovs sites within the ZnO lattice. Conversely, Cr helped produce a CuCr_2_ bimetallic phase within CZC, which inhibited the development of defects in ZnO and countered the promoting effect of Cr^3+^ in the precursor. The interaction between Cu and ZnO was amplified as a result of an increase in Ovs because more electrons were able to pass the Schottky barrier and reach the Cu surface. Consequently, higher Cu^0^/(Cu^0^ + Cu^+^) ratios and Zn^*δ*+^ defect structures were formed. In situ DRIFT results showed that Ovs activated CO to form CO* and CH_*x*_O* intermediates. CH_x_*, formed by CO dissociation and subsequent dissociation or the partial dissociation of CH_*x*_O* at Cu^0^ sites, was involved in C—C coupling with CH_*x*_O*/CO* on Zn^*δ*+^ sites to form ethanol (C_2+_OH). Thus, the CZG catalyst achieved the highest CO conversion (17.22%), with C_2+_OH and ethanol proportions reaching 71.90% and 60.22%, respectively. This study demonstrates that Ovs can be modulated through appropriate promoters to enhance the structural and electronic characteristics of the active sites for C_2+_OH preparation, providing insights for catalyst design and optimization.

## Funding

This study was supported by Taiyuan Institute of Technology Scientific Research Initial Funding (2024KJ031), Scientific Research Award for Outstanding Ph.D. and Postdoct (2025LJ003).

## Conflicts of Interest

The authors declare no conflicts of interest.

## Data Availability

The data that support the findings of this study are available from the corresponding author upon reasonable request.
